# Supplementary material for: Aviation passenger carbon footprint calculator with comprehensive emissions, life cycle coverage, and historical adjustment
Source: Commun Earth Environ. 2025 Oct 29;6(1):855. doi: 10.1038/s43247-025-02847-4 (PMC12576944; doi:10.1038/s43247-025-02847-4)
Supplement: Supplementary file 2 — Supplementary Information [file 43247_2025_2847_MOESM2_ESM.pdf]

Aviation Passenger Carbon Footprint Calculator with Comprehensive Emissions, Life Cycle Coverage, and Historical Adjustment

Finn McFall * [a], *Dabo Guan* [b, c], *Jonathan Chenoweth* [a], *Xavier Font* [d], *Ionut Corduneanu* [e], *Eduard Goean* [d, e], *Jhuma Sadhukhan* [a]

* Corresponding author (f.mcfall@surrey.ac.uk)

[a] Centre for Environment and Sustainability, University of Surrey, Guildford, Surrey, GU2 7XH, UK

[b] The Bartlett School of Construction and Project Management, University College London, London, UK

[c] Department of Earth System Sciences, Tsinghua University, Beijing 100080, China

[d] Surrey Business School, University of Surrey, Guildford, Surrey, GU2 7XH, UK

[e] Therme Group RHTG AG, 1120 Vienna, Austria

1 Supplementary Methods

This section shows the detailed methodology for assessing the carbon footprint of air travel passengers. It improves on existing calculators by introducing variables and refining customary ones for accuracy and comprehensiveness. The result is presented in kilograms of CO_2e, the amount of CO_2 which would have the same global warming potential (GWP) [1]. It incorporates both Kyoto Protocol GHGs in the base methodology, aligning with ISO 14040-44, 14060 series and specifically, 14083, and extended lifecycle impacts such as non-Kyoto substances and infrastructure emissions.

1.1 Goal and Scope Definition

The goal of this study is to deduce the most comprehensive formulations for ATP-DEC to enable per passenger carbon footprint calculation, according to ISO 14083. Because the aviation sector's total environmental footprint spans far beyond the fuel uses, a full cradle-to-grave life cycle model as recommended by ISO 14083 captures hidden CO_2, pollutants, resource depletion, and waste streams that an "in-flight-only" analysis would completely miss and therefore underestimate. By bringing out the life cycle systemic carbon footprint [2,3], actionable offsetting mechanisms can be developed [4]. ATP-DEC's scope is expansive, covering the entire life cycle of emissions. The cradle-to-grave system scope in the aviation sector's context includes the production and transportation of fuel (Well-To-Tank (WTT)), fuel burning during flying, take-off and landing (Tank-To-Wake (TTW)), in-flight services, and airport and aircraft life cycles. As can be seen in Figure 1, TTW mainly contributed by fuel burning, is a small contributor to the overall GWP. The system consideration is the most comprehensive and far beyond the capability of the current aviation sector's carbon footprint calculators.

The ATP-DEC focuses on the life cycle carbon footprint or global warming potential (GWP) estimation in kg CO_2e per passenger, according to ISO 14083. The ATP-DEC's carbon footprint calculation has been broken down into several activities (Table S1) across the aviation sector's life cycles allocated to per flight basis. For each activity, the most comprehensive formulation is developed and GWP characterisation factors were applied accordingly [5], using LCA software SimaPro 9.6. The inventory analysis and impact assessment consist of the formulation developments. In the interpretation, the step-by-step calculation using the ATP-DEC has been demonstrated.

Table S1 outlines the variables incorporated into the tool, and their inclusion in the four existing tools evaluated above.

Table S1: Scope of variables used in this study.

Variable Name	Variable	IATA	ICAO	TIM	MyClimate	ATP-DEC
**Class weighting**	CW	✓	✓	✓	✓	✓
**Passenger load factor**	PLF	✓	✓	✓	✓	✓
**Fuel burn**	$f()$	✓	✓	✓	✓	✓
**TTW emissions factor**	TTW	✓	✓	✓	✓	✓
**Cargo factor**	CF	✓	✓	✓	✓	✓
**Distance**	D		✓	✓	✓	✓
**WTT emissions factor**	WTT			✓	✓	✓
**Aircraft factor**	AF				✓	✓
**Airport factor**	APF			✓	✓	✓
**Non-Kyoto factor**	N					✓
**Deterioration factor**	DT					✓
**Luggage emissions**	L					✓
**In-flight service factor**	IFS					✓

Unlike other tools that focus narrowly on operational emissions, this methodology integrates a broad range of factors to ensure a comprehensive lifecycle assessment. For scientific validity, the entire lifecycle must be considered, regardless of their impact on the final estimation.

1.2 Inventory Analysis and Impact Assessment: Extending the scope of traditional calculators

This section provides the detailed formulations and justification for each activity in Table S1, highlighting their relevance and contribution to a comprehensive aviation sector's carbon footprint assessment. These formulations serve as the basis for inventory data analysis and impact assessment applying GWP factors.

Non-Kyoto Impacts. Nitrogen oxides (NO_x), water vapour (H_2O), contrails and contrail-cirrus cloud formations created by aeroplanes all contribute to aviation's impact on

climate [6]. The proposed methodology adopts the approach suggested by Dahlmann et al (2023), in which they argue that average temperature response is more appropriate for assessing non-Kyoto impact as it accounts for the lifespan of various non-Kyoto substances, the differing climate sensitivities, and the thermal inertia present in the atmosphere-ocean system [7]. The resulting formulas display the relationship of non-Kyoto climate effects on specific mission parameters like distance and geographic flight region. The three non-Kyoto factors included are NO_x, H_2O, and CiC, each with a unique formula. This dynamic model is far superior to a generic Radiative Force Index (RFI) factor. Also, the parameterised model offers a more scientifically robust and comprehensive assessment that simplified methods such as contrail “bucket” classification [8]. The bucket approach provides high-level risk categories based solely on contrail patterns. It omits major contributors like NO_x and water vapour and lacks sensitivity to flight-specific parameters. By failing to convey actual climate impact, it offers no transparent quantification and risks misleading users into underestimating the true scope of non-Kyoto aviation effects.

NO_x gases contribute to the formation of ozone in the troposphere, which is a potent GHG, though ozone itself is not listed in the Kyoto Protocol. H_2O is the most abundant GHG overall, but its concentration in the atmosphere is controlled by the temperature rather than direct human emissions. However, aviation contributes to increased levels of water vapour at high altitudes, amplifying global warming. Contrails from aircraft can evolve into cirrus clouds (CiC) that trap outgoing infrared radiation, contributing to the greenhouse effect. The impact of contrails and induced cloudiness is an area of active research; although it is recognised as having potentially significant climate impacts, there is no consensus way to express that impact to consumers.

The formulas for non-Kyoto impacts were recently developed by Dahlmann et al [7]. The two components for the calculation are distance, D, in 1000km and mean-latitude, L, in degrees. Mean-latitude is the average latitude between the origin and destination airports.

Non-Kyoto impact multipliers are given by,

$$\mathrm{CO_2e^{NO_x}} = (2.3\arctan(3.1D) - 2.0)\left(c_{\mathrm{NO_x}}L^2 + d_{\mathrm{NO_x}}L + e_{\mathrm{NO_x}}\right) \tag{S1}$$

$$\mathrm{CO_2e^{CiC}} = 1.1\arctan(0.5D)\,(a_{\mathrm{CiC}}L^4 + b_{\mathrm{CiC}}L^3 + c_{\mathrm{CiC}}L^2 + d_{\mathrm{CiC}}L + e_{\mathrm{CiC}}) \tag{S2}$$

$$\mathrm{CO_2e^{H_2O}} = 0.2\arctan(D)\,\left(b_{\mathrm{H_2O}}L^3 + c_{\mathrm{H_2O}}L^2 + d_{\mathrm{H_2O}}L + e_{\mathrm{H_2O}}\right) \tag{S3}$$

where a, b, c, d are coefficients computed by Dahlmann et al. [7] (Table S2).

Table S2: Non-Kyoto equation coefficients from Dahlmann et al 7.

Coefficient	NO_x	CiC	H_2O
a	-	2.8×10^{-7}	-
b	-	1.9×10^{-6}	-7.6×10^{-6}
c	1.6×10^{-4}	-1.2×10^{-3}	8.2×10^{-4}
d	-1.6×10^{-3}	-7.7×10^{-4}	1.4×10^{-3}
e	0.86	1.7	0.15

Therefore, each non-Kyoto impact, in kg CO_2e, is computed using:

$$\text{non-Kyoto impact} = \text{CO}_2 \text{ per passenger} \times \text{non-Kyoto multiplier} \tag{S4}$$

Note that all non-Kyoto multipliers are modular and can be separated.

Cargo Factor. Cargo and freight are often carried simultaneously to passengers, so the cargo factor ensures that the environmental impact of the flight is accurately distributed; it acknowledges that not all emissions should be borne by the passenger count alone. This becomes especially important in the application of carbon offsetting or removal programmes.

Luggage Factor and Emissions. Passenger luggage contributes to the overall weight of the aircraft, directly impacting fuel consumption and, consequently, greenhouse gas emissions. The relationship between aircraft weight and fuel burn is well-established: even small changes in weight can lead to measurable differences in emissions [9].

Despite its significance, luggage emissions are often overlooked or generalised in existing carbon calculators. Many tools use an industry-standard passenger and luggage weight of 100kg, which fails to account for variations in luggage weight across classes or baggage allowance policies. This methodology addresses the issue by incorporating a luggage factor and individual luggage emissions. The passenger luggage emissions are separated from the cargo and passenger emissions and split up to be assigned to the passengers.

Both the luggage factor and cargo factor benefit from airline and route-specific passenger, luggage, and cargo weight data. If this is unavailable, the up-to-date 2022 review by EASA and Lufthansa [10] is used to determine the mass of passengers and passenger luggage (Table S3), depending on the plane capacity. The review found the average passenger weight, carry-on luggage, and checked luggage for different classes.

Table S3: Passenger and Luggage Mass by Class (taken from EASA Review, 2022) 10.

Passenger Class	Passenger Mass (kg)	Checked Luggage Mass (kg)	Carry On Luggage (kg)
**First**	80.9	18.7	8.7
**Business**	80.9	18.7	8.7
**Premium Economy**	75.4	17.3	7.6
**Economy**	75.4	17.3	7.6

This can be used to compute the mass of the flight (Equation S5):

$$\text{mass of flight} = \text{PLF}\left(\sum n_s \times m_s\right) + \text{PLF}\left(\sum n_L \times m_L\right) + \left(m_{\text{freight}} + m_{\text{mail}}\right) \tag{S5}$$

where n and m are the number and mass of passenger class (s), passenger class luggage (L), and freight & mail for each flight class.

The passenger luggage factor LF and cargo load factor CF can therefore be calculated using:

$$\text{LF} = 1 - \frac{\text{PLF}(\sum n_L \times m_L)}{\text{mass of flight}} \tag{S6}$$

$$\text{CF} = 1 - \frac{\left(m_{\text{freight}} + m_{\text{mail}}\right)}{\text{mass of flight}} \tag{S7}$$

In-Flight Services. The provision of in-flight services, such as food, beverage and other goods, contributes to the total emissions of a flight passenger. Accounting for these services ensures the methodology encompasses all aspects of the passenger experience. Emissions associated with in-flight services are often overlooked. However, a study by Thrust Carbon found that despite in-flight meal emissions only accounting for ~1% of a traveller's total journey carbon footprint, on a global scale, business travellers' in-flight meals make roughly 564,000 tonnes of CO_2e [11]. Considering a general factor for in-flight services is unique to this methodology. All components of a product, in this case a flight, must be included for comprehensive quantification. For such a small component and one that is inevitably hard to measure, a general factor is acceptable.

Class-specific factors are applied to reflect differences in meal service quality and quantity (e.g. first-class meals vs. economy snacks). Waste factors, such as a 20% food waste [12], are also included.

Airport Factor. In accordance with ISO14083, only the airport (hub) operations must be included in GHG accounting. However, for a comprehensive LCA-based methodology, the construction and maintenance of the airports should be included.

Ecoinvent provides a value for the kg CO_2e of the construction and usage of an airport dependent on the distance flown. Their comprehensive assessment is based on Zurich airport, classed as a medium sized airport. It is assumed that each passenger uses two airports, and the lifetime of an airport is 100 years. Table S4 shows the Ecoinvent 3.10 kg CO_2e values per kilometre (km) flown within the corresponding flight distance range.

Ecoinvent is a globally recognised life cycle inventory (LCI) database that provides comprehensive, standardised, and peer-reviewed datasets essential for robust life cycle assessment (LCA). Its use ensures consistency and reliability, enabling accurate environmental impact characterisations critical for systems, which involve complex, interconnected processes. By offering validated data, Ecoinvent addresses the key challenge of data availability in LCA, supporting replicable, transparent, and scientifically credible assessments necessary for evaluating carbon impacts and guiding sustainability strategies [13,14].

Aircraft Factor. The lifecycle of an aircraft includes its construction, maintenance, and disposal. Ecoinvent provides the CO_2e impact of aircraft construction and maintenance, averaged over the construction estimates of forty different aircrafts.

Table S4: Airport and aircraft factors by distance, derived from Ecoinvent 3.10.

Flight distance	Airport factor (kg CO_2e per km)	Aircraft factor (kg CO_2e per km)
**D < 800km**	0.0208	0.000267
**800km < D < 1500km**	0.0109	0.000252
**1500km < D < 4000km**	0.00455	0.000236
**D > 4000km**	0.00157	0.000144

2.1. Inventory Analysis and Impact Assessment: Accuracy

This section showcases the high level of accuracy achieved by ATP-DEC, combining dynamic models, specific inputs, and lifecycle data to deliver exceptional precision.
1.2.1It summarises the method and accuracy of key factors in existing tools,
1.2.2presents accuracy improvements on traditional factors in carbon calculators,
1.2.3and explains how accuracy is further enhanced using historical flight data.

1.2.1 Accuracy level of existing tools and ATP-DEC

Table S5 shows a comparison of methods and accuracy for key variables between existing tools and ATP-DEC's advanced methodology.

Table S5: Methods and accuracy comparison of key factors in existing tools and ATP-DEC.

<table>
<tr><th>Variable Name</th><th>Variable</th><th>IATA</th><th>ICAO</th><th>TIM</th><th>MyClimate</th><th>ATP-DEC</th></tr>
<tr><td>Distance</td><td>D</td><td>Mid: Time-based method</td><td>Low: GCD</td><td colspan="2">Low-mid: GCD with generalised distance adjustment</td><td>High: Historically adjusted route and airline-specific distance</td></tr>
<tr><td>Fuel burn</td><td>$f()$</td><td colspan="3">Mid: Linearly interpolated fuel burn model</td><td>Mid: Generalised polynomial function</td><td>Mid-high: Polynomial regression fuel interpolation model</td></tr>
<tr><td>TTW emissions factor</td><td>TTW</td><td colspan="4">Mid: Non-fuel-specific TTW</td><td>Mid-high: Option for fuel-specific TTW</td></tr>
<tr><td>WTT emissions factor</td><td>WTT</td><td colspan="2">N/a</td><td colspan="2">Mid: Non-fuel-specific WTT</td><td>Mid-high: Option for fuel-specific WTT</td></tr>
<tr><td>Deterioration factor</td><td>DT</td><td colspan="4">N/a</td><td>Mid: Age-dependent DT</td></tr>
<tr><td>Class weighting</td><td>CW</td><td colspan="4">Low: Generalised IATA constants</td><td>High: Area-based CW using aircraft-specific seat configurations</td></tr>
<tr><td>Passenger load factor</td><td>PLF</td><td colspan="4">Mid: Generalised PLF</td><td>High: Historcially adjusted route and airline-specific PLF</td></tr>
<tr><td>Cargo factor</td><td>CF</td><td colspan="2">High: Weight-based CF</td><td>N/a</td><td>Low-mid: Weight-based with average load constant</td><td>High: Weight-based CF</td></tr>
<tr><td>Luggage emissions</td><td>L</td><td colspan="4">N/a</td><td>High: Individual weight and quantity-specific luggage emissions</td></tr>
<tr><td>In-flight service factor</td><td>IFS</td><td colspan="4">N/a</td><td>Mid-high: Route and airline-specific IFS</td></tr>
<tr><td>Airport factor</td><td>APF</td><td colspan="3">N/a</td><td>Mid: Generalised constant</td><td>Mid-high: Ecoinvent 3.10 flight distance-based factors</td></tr>
<tr><td>Aircraft factor</td><td>AF</td><td colspan="3">N/a</td><td>Mid: Generalised constant</td><td>Mid-high: Ecoinvent 3.10 flight distance-based factors</td></tr>
<tr><td>Non-Kyoto factor</td><td>N</td><td colspan="2">N/a</td><td>Low: Over-simplified buckets</td><td>Low: Generalised constant RFI value</td><td>High: Dynamic accurate non-Kyoto model</td></tr>
<tr><td>Historical Adjustment Factors (HAF)</td><td>$\mathcal{G}, \mathcal{V}, \mathcal{Z}_{1,2}$</td><td colspan="4">N/a</td><td>High: Derived from route, airline and aircraft-specific historical data</td></tr>
</table>

1.2.2 Accuracy improvement of traditional factors

Distance. Initially, the flight distance is determined using the Great Circle Distance (GCD). HAF are used to dynamically adjust the distance based on historical flight data (Equation S8). In cases where HAF isn't available due to lack of data, the GCD is supplemented by a Distance Adjustment Factor (DAF) to account for route deviations and air traffic control constraints (Equation S8).

$$D = \begin{cases} \mathrm{GCD} \times \mathrm{HAF}, & \text{if historical data exists} \\ \mathrm{GCD} \times \mathrm{DAF}, & \text{otherwise} \end{cases} \tag{S8}$$

Where the DAF is calculated from the results of a study conducted by Dobruzkes & Peeters (2019). This dynamic adjustment improves precision compared to static correction factors adopted by existing calculators.

Fuel Burn. Fuel consumption is divided into two components:

- Landing and Take-Off (LTO): encompasses the taxi-in, taxi-out, take-off, climb-out, approach, and landing.
- Cruise, Climb, Descent (CCD): CCD includes the fuel burn at altitude.

The total fuel burn is computed by summing the LTO and CCD fuel burn together. Since the relationship between distance flown and fuel burn is marginally non-linear (fuel is used up during flight, making the aircraft lighter), a polynomial regression model is fitted and used to predict the fuel burn for distances in between two data points or extrapolate for distances outside the data range.

Before converting to emissions, a deterioration factor is applied to the fuel consumption to account for aircraft inefficiencies. Deterioration factor is dependent on the age of the aircraft. If the aircraft age is unknown, the age of the aircraft is assumed to be 10+ years old, and the maximum deterioration factor is applied. This is based off data provided by IATA, which states the global average aircraft age is 11.6 years old [15].

Fuel burn emissions are split into Tank-to-Wake (TTW) and Well-to-Tank (WTT) components.

Tank-to-Wake (TTW) emissions refer to the direct emissions released during the combustion of aviation fuel in flight. This factor forms the largest share of a flight's operational emissions. The type of aviation fuel used greatly influences fuel consumption and emissions. Commercial aircraft primarily utilise Jet A or Jet A-1 fuels, kerosene-based fuels known for their high energy content and efficiency. ISO 14083 give a TTW emissions factor of 3.16kg CO_2e. However, it's vital to incorporate a capability to utilise fuel-specific emission factors. Unique to this method, enabling fuel-specific emissions factors are essential for promoting the use of SAF.

Well-to-Tank (WTT) emissions account for the upstream emissions associated with the production, refining, and transportation of aviation fuel. These emissions occur before the fuel reaches the aircraft and are critical for a full lifecycle assessment. The distinction between upstream and combustion emissions ensures transparency and enables the tool to reflect the environmental benefits of cleaner fuel technologies. ISO 14083 give a TTW emissions factor of 0.48kg CO_2e.

Passenger Load Factor. The GHG emissions per aircraft are distributed across all passengers. A flight with high occupancy is more efficient on a per-passenger basis than one with many empty seats, highlighting the importance of load factors in environmental impact assessments. Most existing calculators incorporate passenger load factors to account for this efficiency, recognising it as a crucial component in accurately determining per-passenger emissions. However, many of these calculators often rely on a generalised constant PLF, which does not account for seasonal variations, route-specific demand fluctuations, or airline-specific operational practices that influence occupancy. To address these shortcomings, the proposed methodology will base load factor calculations on historical passenger load factor figures specific to each flight path and airline. In cases where historical PLF load factors are unavailable, a value of 0.832 will be used (83.2%), derived from the US Bureau of Transportation Statistics 2023 average [16].

Class Weighting. The class weighting variable adjusts for the disproportionate space used by different classes of seating. Premium seating often occupies more space, thus contributing to a greater carbon footprint per passenger compared to economy seating [17]. IATA proposes generalised seating class weights, which are used by almost all existing calculators. Whilst convenient, this approach lacks the precision necessary to account for the variations in aircraft configurations and the specific layout of individual airlines. The methodology proposed here adopts a more accurate and credible approach, in which the seating class width and pitch are assessed for each aircraft seating configuration.

Given the number, width, and pitch of each seat class (first, business, premium economy, economy), the total seat area is computed using,

$$\text{total seat area} = \sum(n_s \times w_s \times p_s) \tag{S9}$$

where n_s, w_s, p_s are the number, width, and pitch of each seat class, s. This allows for the calculation of accurate class weighting. For each class, the class weighting is given by,

$$\text{class weighting} = \frac{w_s \times p_s}{\text{total seat area}} \tag{S10}$$

When seat area data is unavailable for a flight, cabin class factors provided by IATA RP 1726. These values are treated as seat areas, equivalent to $w_s \times p_s$ in the method above.

The CO_2e per passenger in each class is the product of total CO_2e and the class weight:

$$\mathrm{CO_2e\ per\ class} = \mathrm{total\ CO_2e} \times \mathrm{class\ weighting} \tag{S11}$$

1.2.3 Extended accuracy using historical adjustment factors

Historical Adjustment Factors. To enhance the accuracy of pre-flight emissions estimates, a historical adjustment factor (HAF) is calculated and applied to estimated variables. An HAF is a value calculated using the comparison of post-flight and pre-flight emissions data from a series of past flights. This scalar accounts for deviations and variances observed in actual flight operations, thereby refining future emissions calculations.

An HAF, is calculated as the average of the ratio error:

$$\mathrm{HAF} = \frac{1}{n}\sum_{i=1}^{n} \frac{\text{post-flight estimate}_{\mathrm{i}}}{\text{pre-flight estimate}_{i}} \tag{S12}$$

The letter i represents a single historical estimate, where $i = 1$ is the most recent historical estimate (excluding the current estimate) and $i = n$ is the final historical estimate from the most recent n flights.

Dependent on available data, the value of n should be determined using machine learning (ML) techniques such as reinforcement learning or Bayesian optimisation.

The HAF only adjusts the estimated components within the formula: distance and fuel burn, $f(\mathrm{D})$, cargo factor, CF, passenger load factor, PLF, in-flight service, IFS, and non-Kyoto impacts, N, broken down into two scalar HAFs for $f(\mathrm{D})$ and mean latitude, Lat. The HAFs within the formula are Geta, $\mathcal{G}$, Veta, $\mathcal{V}$, and Zeta, $\mathcal{Z}_{1,2}$:

$$\mathcal{G} = \frac{\mathrm{HAF}_{f(\mathrm{D})} \times \mathrm{HAF}_{\mathrm{CF}}}{\mathrm{HAF}_{\mathrm{PLF}}} \tag{S13}$$

$$\mathcal{V} = \mathrm{HAF}_{\mathrm{IFS}} \tag{$S14$}$$

$$\mathcal{Z}_{1,2} = \frac{\mathrm{HAF}_{f(\mathrm{D})} \times \mathrm{HAF}_{\mathrm{CF}}}{\mathrm{HAF}_{\mathrm{PLF}}}, \mathrm{HAF}_{Lat} \tag{$S15$}$$

The nonlinear characteristics of the formula mean that adjusting individual variables according to their specific changes is more accurate for predictive purposes, as opposed to adjusting the whole formula with one factor.

1.3 Interpretation: ATP-DEC's step-by-step calculations

This section summarises the step-by-step calculations to interpret the results, including hotspot analysis (Figure 1).

$$\mathrm{CO_2e\ per\ pax} = \mathcal{G}\left(\frac{f(\mathrm{D}) \times (\mathrm{WTT{+}TTW}) \times \mathrm{DT} \times \mathrm{CF} \times \mathrm{LF} \times \mathrm{CW}}{\mathrm{PLF}}\right) + L + (\mathcal{V} \times \mathrm{IFS}) + \mathrm{APF} + \mathrm{AF} + (\mathcal{Z}_1 \times \mathrm{N}(\mathcal{Z}_2)) \quad (S16)$$

For steps 1.3.1 to 1.3.6 the scope aligns with ISO14083 and provides a robust, standardised framework for emission calculations. The extended methodology, 1.3.7 to 1.3.9, supplements the method, elevating comprehensiveness. The additional and enhanced factors within the extended methodology go beyond the scope of ISO14083 and are optional. The ATP-DEC methodology outlines a full, holistic, modular formula that acknowledges the importance of a thorough environmental assessment while allowing flexibility for the stakeholder to encourage environmental action.

Illustrative calculations for a flight from Singapore to Zurich (Figure 1) are implemented along with each step.

1.3.1 Calculate distance

Calculate the initial distance using the GCD and DAF (Equation S8). For Figure 1 (Singapore to Zurich) the GCD is 10309km, calculated using the latitude and longitude (ϕ, γ) of the airports in degrees and the earth radius (r) substituted into the Haversine formula (Equation S17, S18).

$$\mathrm{GCD} = 2r \arcsin\left(\sqrt{\frac{1 - \cos(\phi_2 - \phi_1) + \cos(\phi_1) \cdot \cos(\phi_2) \cdot (1 - \cos(\gamma_2 - \gamma_1))}{2}}\right) \quad (\mathrm{S17})$$

$$\mathrm{GCD} = 2(6372) \arcsin\left(\sqrt{\frac{1 - \cos(0.85 - 1.36) + \cos(1.36) \cdot \cos(0.85) \cdot (1 - \cos(0.15 - 1.81))}{2}}\right) \quad (\mathrm{S18})$$

An HAF can be used to adjust this to a more precise flight distance (Equation S13). For Figure 1, historical flight data from the arbitrarily chosen final week in December 2019 was used to calculate a distance HAF of 1.0315 (Equation S12). For example, if 2 successive flight distances in December are substituted into Equation S12:

$$\mathrm{HAF} = \frac{1}{n}\sum_{i=1}^{n} \frac{\text{post-flight estimate}_{\mathrm{i}}}{\text{pre-flight estimate}_i} = \frac{1}{2}\sum \frac{10680}{10309} + \frac{10676}{10309} = 1.0358 \quad (\mathrm{S19})$$

This example (Equation S19) outputs a higher HAF than 1.0315, which is why it's important to calculate HAF over an extended period (Table 1). The HAF can be updated with a rolling window of 7 or 30 days (Table 1). A HAF of 1.0315 means that the average distance of a flight in this month was 1.0315 times higher than the GCD and we use this to predict the distance of the flight example in Figure 1 as 10634km.

$$\mathrm{D} = 10309 \times 1.0315 = 10634\mathrm{km}$$

1.3.2 Extract fuel burn

Use the polynomial regression model alongside the fuel burn data to equate the distance flown with the aircraft-specific fuel consumption. Include both the LTO and CCD fuel consumption. For the results in this study, Eurocontrol fuel data is used with the polynomial regression model. Fuel burn, including CCD and LTO, for 10634km for a Boeing 777-300 (B773) is 89540kg (this is the manuscript example in Figure 1). The polynomial model for the CCD fuel burn is represented in Figure S1. Apply a deterioration factor depending on the age of the engine within the aircraft. For a wide-bodied B773 with an age of 1 year, the deterioration factor is 1.01, so the fuel consumption is given as 90435kg (Figure 1).

Figure S1: Polynomial regression model for the fuel burn of Boeing 777-300

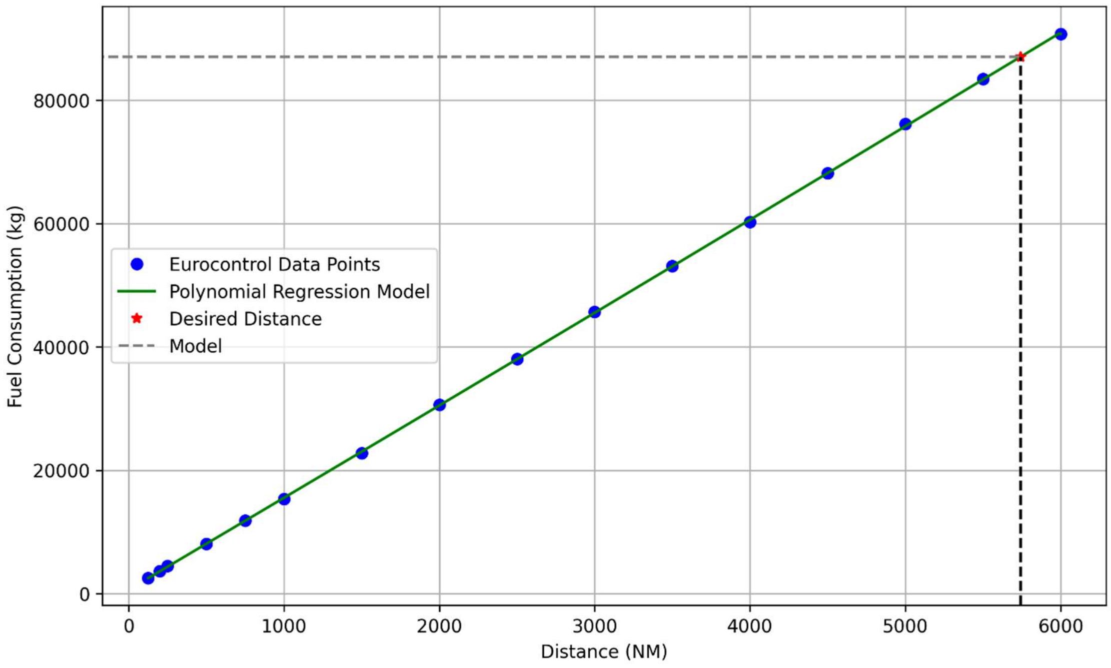

1.3.3 Compute total aircraft emissions

Multiply the fuel consumption with the WTT and TTW emissions factors and sum them together to calculate the total emissions for the flight. This includes all passenger, passenger luggage, and cargo emissions. WTT for 90435kg of Jet A1 fuel is 43409kg CO_2e (Equation S20) and TTW is 285776kg CO_2e (Equation S21). This is calculated using emission factors from ISO 14083 [18].

$$\text{WWT} = 90435 \times 0.48 = 43409\text{kg} \tag{S20}$$

$$\text{TTW} = 90435 \times 3.16 = 285776\text{kg} \tag{S21}$$

1.3.4 Adjust for cargo and passenger luggage allocation

For emission accounting, the total mass of the aircraft can be assumed to be the sum of passengers, passenger luggage, and cargo (freight and mail). After subsequently calculating the cargo and luggage factors, the total emissions are adjusted to account solely for passengers.

The total mass of the example flight (Figure 1) is computed by summing the mass of passengers, passenger luggage, and cargo (Equation S5). SeatGuru data was used for the seating configuration (Table S6) and a PLF of 85%. For fair comparison, cargo mass is assumed to be zero for this flight because other tools did not consider cargo allocation.

$$\text{mass of passengers} = 0.85((4 \times 80.9) + (48 \times 80.9) + (28 \times 75.4) + (184 \times 75.4)) = 17163\text{kg} \quad (S22)$$

$$\text{mass of carry-on luggage} = 0.85((4 \times 8.7) + (48 \times 8.7) + (28 \times 7.6) + (184 \times 7.6)) = 1754\text{kg} \quad (S23)$$

$$\text{mass of checked luggage} = 0.85((4 \times 18.7) + (48 \times 18.7) + (28 \times 17.3) + (184 \times 17.3)) = 3944\text{kg} \quad (S24)$$

$$\text{mass of cargo} = 0\text{kg} \quad (S25)$$

$$\text{total mass of flight} = 17163 + 1754 + 3944 + 0 = 22861\text{kg} \quad (S26)$$

Luggage factors and cargo factor are calculated as follows:

$$LF_{\text{carry-on}} = 1 - \frac{1754}{22861} = 0.923\ldots \quad (S27)$$

$$LF_{\text{checked}} = 1 - \frac{3994}{22861} = 0.827\ldots \quad (S28)$$

$$\text{CF} = 1 - 0 = 1 \quad (S29)$$

By separating these factors (Equation S27, S28, S29), ATP-DEC formulates a full breakdown ratio of emissions for the flight in terms of passengers to carry-on luggage to checked luggage to cargo factor.

After adjusting for cargo and luggage masses (Table S3), the total passenger fuel consumption emissions come out at 251111kg CO_2e (Equation S30, S31).

$$\begin{aligned}\text{Total passenger WTT} &= \text{WTT} \times \text{LF}_{\text{carry-on}} \times \text{LF}_{\text{checked}} \times \text{CF} \\ &= 43409 \times 0.92 \times 0.83 \times 1 = 33147 \quad (S30)\end{aligned}$$

$$\begin{aligned}\text{Total passenger TTW} &= \text{TTW} \times \text{LF}_{\text{carry-o}} \times \text{LF}_{\text{checked}} \times \text{CF} \\ &= 285776 \times 0.92 \times 0.83 \times 1 = 218219 \quad (S31)\end{aligned}$$

1.3.5 Compute class weightings

Class weighting is calculated by dividing the area of a single seat in each class by the total seat area of the aircraft (Equation S10). The table below (Table S6) represents the seat configuration data utilised for the Singapore to Zurich example (Figure 1).

Table S6: Seating configuration for Boeing 777-300 example from SeatGuru (Figure 1).

Passenger Seat Class	Number	Pitch (cm)	Width (cm)
**First**	4	81	35
**Business**	48	55	28
**Premium Economy**	28	38	19.5
**Economy**	184	32	19

Substituting the number, pitch, and width of each seat class into Equation S9 gives a total seat area of 217880cm^2. For example, the class weighting for a First-Class passenger on the Boeing 777-300 example (Figure 1) is calculated by substituting into the First-Class pitch p_1, width w_1, and total seat area Equation S10.

$$\text{first class weighting} = \frac{w_1 \times p_1}{\text{total seat area}} = \frac{35 \times 81}{217880} = 0.01301 \tag{S32}$$

The same procedure is used to calculate each passenger class (Table S7).

Table S7: Passenger seat class weightings for Boeing 777-300 example (Figure 1).

Passenger Seat Class	Weighting
**First**	0.01301
**Business**	0.00707
**Premium Economy**	0.00340
**Economy**	0.00279

The WTT and TTW emissions are multiplied by the class weighting to compute the mass of CO_2e for each passenger in each class for a fully occupied flight (Equation S11) (Table S8) (Figure 1).

Table S8: Per passenger WTT and TTW emissions by passenger seat class.

Passenger Seat Class	WTT emissions per passenger for fully occupied example flight (kg CO_2e)	TTW emissions per passenger for fully occupied example flight (kg CO_2e)
**First**	431.30	2893.41
**Business**	234.29	1542.39
**Premium Economy**	112.73	742.15
**Economy**	92.50	608.94

1.3.6 Adjust for empty seats (PLF)

The GHG emissions per passenger are determined by dividing the total passenger allocated GHG emissions, E, by the passenger load factor, PLF , of the flight. The PLF accuracy can be enhanced with historical data. The estimated occupancy for the

example Singapore to Zurich flight is 85% (Figure 1) so the PLF is 0.85. Table S9 shows the per passenger WTT and TTW emissions adjusted for the correct occupancy (Figure 1).

Table S9: Per passenger WTT and TTW emissions by passenger seat class for example flight (Figure 1).

Passenger Seat Class	WTT emissions per passenger for 85% occupied example flight (kg CO_2e)	TTW emissions per passenger for 85% occupied example flight (kg CO_2e)
**First**	499.11	3285.82
**Business**	271.12	1784.89
**Premium Economy**	130.46	858.83
**Economy**	107.04	704.68

1.3.7 Calculate passenger luggage emissions

Total passenger luggage allocated CO_2e emissions is given by removing the passenger and cargo emissions. This separation allows for individual calculation of luggage carbon footprint. The total passenger luggage allocated CO_2e emissions is split up into passenger classes using the airline and route-specific passenger and luggage weight data. If unavailable, EASA data provides average weights of carry-on and checked luggage for passengers divided by class. Economy and premium economy passenger luggage weighs roughly 91% that of business and first passengers.

As the individual weight of luggage is known, carry-on and checked luggage emissions can be calculated separately to passengers, contributing the overall breakdown. For the Singapore to Zurich example flight (Figure 1), utilising EASA data [10], the carry-on and checked luggage emissions are calculated independently to the passenger emissions. This allows passengers to select the luggage that they wish to take and can enforce more sustainable choices. Table S10 shows these emissions. It’s important to note that the carry-on and checked luggage emissions in this table represents the full comprehensive output, including non-Kyoto impacts as well as WTT and TTW (Figure 1).

Table S10: Passenger luggage emissions for example flight (Figure 1). Emission values include non-Kyoto impacts.

Passenger Seat Class	Carry-on luggage emissions per passenger for example flight (kg CO_2e)	Checked luggage emissions per passenger for example flight (kg CO_2e)
**First**	314.70	676.43
**Business**	314.70	676.43
**Premium Economy**	274.91	625.78
**Economy**	274.91	625.78

1.3.8 Add in-flight service, airport and aircraft factors

Add the in-flight service constant dependent on the passenger class and add the airport and aircraft constants dependent on the distance of the flight (Table S4).

1.3.9 Non-Kyoto impacts

The output of the non-Kyoto formulae is multiplied by the CO_2 emissions per passenger, which must be calculated individually, using a very similar method to steps 3.3.1 to 3.3.6, replacing the WTT and TTW emissions factors with the CO_2 emissions factor of 3.15kg CO_2 per kg of fuel [7]. This step is taken to prevent double counting of emissions, since Kyoto gases must be removed for the non-Kyoto impact of NO_x, H_2O, CiC.

For the Singapore to Zurich example, the HAF-refined distance is 10634km. Converted to thousand kilometres, for D in the non-Kyoto equations, this is 10.634. The mean latitude, L, between the airports is 24.4° but the HAF-refined mean latitude is 28.4° based on historical operational latitude data.

These values are substituted into the non-Kyoto equations (Equation S1, S2, S3) [7] to compute the non-Kyoto multipliers of 1.46, 1.42, and 0.20 for NO_x, CiC, and H_2O respectively (Equation S33, S34, S35).

$$\begin{aligned} \mathrm{CO_2e^{NO_x}} &= (2.3\arctan(3.1D) - 2.0)\left(c_{\mathrm{NO_x}}L^2 + d_{\mathrm{NO_x}}L + e_{\mathrm{NO_x}}\right) \\ &= (2.3\arctan(3.1(10.634) - 2.0)\left(c_{\mathrm{NO_x}}28.4^2 + d_{\mathrm{NO_x}}28.4 + e_{\mathrm{NO_x}}\right) \\ &= 1.46 \end{aligned} \tag{S33}$$

$$\begin{aligned} \mathrm{CO_2e^{CiC}} &= 1.1\arctan(0.5D)\left(a_{\mathrm{CiC}}L^4 + b_{\mathrm{CiC}}L^3 + c_{\mathrm{CiC}}L^2 + d_{\mathrm{CiC}}L + e_{\mathrm{CiC}}\right) \\ &= 1.1\arctan(0.5(10.634))\left(a_{\mathrm{CiC}}28.4^4 + b_{\mathrm{CiC}}28.4^3 + c_{\mathrm{CiC}}28.4^2 + d_{\mathrm{CiC}}28.4 + e_{\mathrm{CiC}}\right) \\ &= 1.42 \end{aligned} \tag{S34}$$

$$\begin{aligned} \mathrm{CO_2e^{H_2O}} &= 0.2\arctan(D)\left(b_{\mathrm{H_2O}}0.48^3 + c_{\mathrm{H_2O}}0.48^2 + d_{\mathrm{H_2O}}0.48 + e_{\mathrm{H_2O}}\right) \\ &= 0.2\arctan\left((10.634)\right)\left(b_{\mathrm{H_2O}}28.4^3 + c_{\mathrm{H_2O}}28.4^2 + d_{\mathrm{H_2O}}28.4 + e_{\mathrm{H_2O}}\right) \\ &= 0.20 \end{aligned} \tag{S35}$$

Multiplying by the per passenger CO_2 gives the non-Kyoto impact for each class (Equation S4) (Table S11).

Table S11: Non-Kyoto impacts per passenger for each passenger class for Singapore to Zurich example flight (Figure 1).

Passenger Seat Class	NO_x emissions per passenger for example flight (kg CO_2e)	CiC emissions per passenger for example flight (kg CO_2e)	H_2O emissions per passenger for example flight (kg CO_2e)
**First**	4770.12	4665.48	655.77
**Business**	2491.17	2534.33	356.22
**Premium Economy**	1246.79	1219.44	171.40
**Economy**	1023.01	1000.58	140.64

For the full emissions breakdown of this case study calculation results, see Figure 1.

1.4 Results Set Up

To validate the ATP-DEC model, and to assess the effectiveness of the HAF algorithm under varying geopolitical and operational conditions, a targeted selection of flight routes was required. Historical operational flight data was acquired commercially from FlightRadar24, and the validation strategy centred on comparing flight behaviours before and after a major global aviation event: the closure of Russian airspace to most international carriers in early 2022. This event represented a well-defined disruption to global flight routing, particularly for long-haul flights between Europe and Asia. To isolate the effects of this airspace restriction and avoid confounding impacts from the COVID-19 pandemic, we selected two time periods for comparison – 2019 (pre-pandemic and pre-airspace closure) and 2023 (post-closure with stable operational patterns). Six long-haul routes known to historically traverse Russian airspace were chosen to serve as case studies. These routes were particularly well suited for validating HAF performance, as they exhibit clear and measurable shifts in actual flight distance and trajectory due to rerouting. By evaluating ATP-DEC's predictive capability against this data, we were able to rigorously test the model's responsiveness to real-world variation and ensure its generalisability across different temporal and geopolitical contexts.

2 Supplementary References

1 Brander, M. & Davis, G. *Greenhouse gases, CO2, CO2e, and carbon: What do all these terms mean*, <https://bluemangrove.fund/wp-content/uploads/2021/03/Glossary-on-different-CO2-terms.pdf> (2012).

2 Sadhukhan, J. Net zero electricity systems in global economies by life cycle assessment (LCA) considering ecosystem, health, monetization, and soil CO2 sequestration impacts. *Renewable Energy* **184**, 960-974 (2022). https://doi.org/10.1016/j.renene.2021.12.024

3 Rupcic, L. *et al.* Environmental impacts in the civil aviation sector: Current state and guidance. *Transportation Research Part D: Transport and Environment* **119**, 103717 (2023). https://doi.org/10.1016/j.trd.2023.103717

4 Goean, E. R. *et al.* Using the Blockchain to Reduce Carbon Emissions in the Visitor Economy. *Sustainability* **16**, 4000 (2024). https://doi.org/10.3390/su16104000

5 IPCC. Climate Change 2023: Synthesis Report. Contribution of Working Groups I, II and III to the Sixth Assessment Report of the Intergovernmental Panel on Climate Change. 35-115 (IPCC, Geneva, Switzerland, 2023).

6 Lee, D. S. *et al.* The contribution of global aviation to anthropogenic climate forcing for 2000 to 2018. *Atmospheric environment* **244**, 117834 (2021). https://doi.org/10.1016/j.atmosenv.2020.117834

7 Dahlmann, K., Grewe, V., Matthes, S. & Yamashita, H. Climate assessment of single flights: Deduction of route specific equivalent CO2 emissions. *International Journal of Sustainable Transportation* **17**, 29-40 (2023). https://doi.org/10.1080/15568318.2021.1979136

8 Travel Impact Model (TIM) v. 3.0.0 (Google, 2022).

9 Zheng, X. S. & Rutherford, D. *Fuel burn of new commercial jet aircraft: 1960 to 2019*, <https://theicct.org/wp-content/uploads/2021/06/Aircraft-fuel-burn-trends-sept2020.pdf> (2020).

10 Harald, B. *et al.* *Review of Standard Passenger Weights*, <https://www.easa.europa.eu/sites/default/files/dfu/EASA%20Final%20Report%20-%20Review%20of%20standard%20passenger%20weights.pdf> (2022).

11 Bonaiuto, A. *From the Sky to the Plate: Thrust Carbon Unveils Carbon Emissions Data for In-Flight Meals*, <https://thrustcarbon.com/insights/from-the-sky-to-the-plate-thrust-carbon-unveils-carbon-emissions-data-for-in-flight-meals?utm_source=website&utm_medium=blog&utm_campaign=meal%20emissions> (2024).

12 IATA. *IATA Cabin Waste Handbook*, <https://www.iata.org/contentassets/821b593dd8cd4f4aa33b63ab9e35368b/iata-cabin-waste-handbook.pdf> (2017).

13 Sadhukhan, J. & Sen, S. A novel mathematical modelling platform for evaluation of a novel biorefinery design with Green hydrogen recovery to produce renewable aviation fuel. *Chemical Engineering Research and Design* **175**, 358-379 (2021). https://doi.org/10.1016/j.cherd.2021.09.014

14 Sadhukhan, J., Sen, S. & Gadkari, S. The mathematics of life cycle sustainability assessment. *Journal of Cleaner Production* **309**, 127457 (2021). https://doi.org/10.1016/j.jclepro.2021.127457

15 IATA. *Balancing fleet age for efficiency and sustainable growth*, <https://www.iata.org/en/iata-repository/publications/economic-reports/chart-of-the-week-8-sep/> (2023).

16 U.S. Bureau of Transportation Statistics. *Load Factor for U.S. Air Carrier Domestic and International*, <https://fred.stlouisfed.org/series/LOADFACTORD11> (2023).

17 Park, Y. & O'Kelly, M. E. Fuel burn rates of commercial passenger aircraft: variations by seat configuration and stage distance. *Journal of transport geography* **41**, 137-147 (2014). https://doi.org/10.1016/j.jtrangeo.2014.08.017

18 International Organization for Standardization. Vol. ISO 14083:2023 117 pp (ISO, Geneva, Switzerland, 2023).
